# HLA‐G whole gene amplification reveals linkage disequilibrium between the HLA‐G 3′UTR and coding sequence

**DOI:** 10.1111/tan.13909

**Published:** 2020-05-17

**Authors:** Jos J. M. Drabbels, Robert Welleweerd, Inge van Rooy, Guro M. Johnsen, Anne Cathrine Staff, Geert W. Haasnoot, Nienke Westerink, Frans H. J. Claas, Erik Rozemuller, Michael Eikmans

**Affiliations:** ^1^ Department of Immunohematology Leiden University Medical Center Leiden The Netherlands; ^2^ GenDx Utrecht The Netherlands; ^3^ Institute for Clinical Medicine, Faculty of Medicine University of Oslo Oslo Norway; ^4^ Division of Obstetrics and Gyneacology Oslo University Hospital Oslo Norway

**Keywords:** 3′‐UTR, allele, haplotype, HLA‐G, next generation sequencing

## Abstract

Polymorphic sites in the HLA‐G gene may influence expression and function of the protein. Knowledge of the association between high‐resolution HLA‐G alleles and 3‐prime untranslated (3′UTR) haplotypes is useful for studies on the role of HLA‐G in transplantation, pregnancy, and cancer. We developed a next generation sequencing (NGS)‐based typing assay enabling full phasing over the whole HLA‐G gene sequence with inclusion of the 3′UTR region. DNA from 171 mother‐child pairs (342 samples) was studied for: (a) HLA‐G allele information by the NGSgo‐AmpX HLA‐G assay, (b) 3′UTR haplotype information by an in‐house developed sequence‐based typing method of a 699/713 base pair region in the 3′UTR, and (c) the full phase HLA‐G gene sequence, by combining primers from both assays. The mother to child inheritance allowed internal verification of newly identified alleles and of association between coding and UTR regions. The NGSgo workflow compatible with Illumina platforms was employed. Data was interpreted using NGSengine software. In 99.4% of all alleles analyzed, the extended typing was consistent with the separate allele and 3′UTR typing methods. After repeated analysis of four samples that showed discrepancy, consistency reached 100%. A high‐linkage disequilibrium between IPD‐IMGT/HLA Database‐defined HLA‐G alleles and the extended 3′UTR region was identified (*D*′ = 0.994, *P* < .0001). Strong associations were found particularly between HLA‐G*01:04 and UTR‐3, between HLA‐G*01:01:03 and UTR‐7, and between HLA‐G*01:03:01 and UTR‐5 (for all: *r* = 1). Six novel HLA‐G alleles and three novel 3′UTR haplotype variants were identified, of which three and one, respectively, were verified in the offspring.

## 1 BACKGROUND

The human leukocyte antigen‐G (HLA‐G) is a non‐classical major histocompatibility complex class Ib glycoprotein, which can have immune regulatory properties. HLA‐G was first described in the placenta.^1^ For healthy pregnancy, the immune system of the mother needs to accept the semi‐allogeneic fetal tissue. HLA‐G is expressed by fetal extravillous trophoblasts in the placenta where it is thought to be involved in reinforcing immune tolerance of maternal immune cells toward the fetus.^2^, ^3^ Low levels of soluble HLA‐G have been associated with pregnancy complications.^4^, ^5^, ^6^, ^7^ HLA‐G may be involved also in mediating mechanisms for immune tolerance and allograft acceptance after transplantation,^8^, ^9^ and in immune escape mechanisms in tumors.^9^

In contrast to classical HLA genes (HLA‐A, ‐B, ‐C), HLA‐G is far less polymorphic. An extensive study has been published on the HLA‐G gene structure and haplotype diversity in populations over the world.^10^ Both genetic variations in the coding sequence and in the 3 prime untranslated region (3′UTR) of the HLA‐G gene may affect the level and function of the gene product. Certain HLA‐G genotype variants are associated with soluble (s) HLA‐G levels in the plasma^11^ and functionality of the protein.^12^ The 3′UTR is targeted by microRNAs,^13^ which can mediate reduced expression of the mRNA and/or protein. Polymorphisms in this 3′UTR region, such as the one at +3142,^14^ may influence the efficiency of microRNA binding, and consequently the level of HLA‐G expression. The 14‐bp insertion/deletion and +3187A polymorphisms influence the stability of the HLA‐G mRNA molecule and the expression of HLA‐G.^15^, ^16^, ^17^ Castelli and colleagues composed 3′UTR haplotypes on the basis of eight different polymorphisms in the HLA‐G 3′UTR region.^18^ Several studies have shown that the 3′UTR haplotype is related to (soluble) HLA‐G levels.^19^, ^20^

Next generation sequencing methods have made it easier to obtain nucleotide sequence information of the full HLA‐G gene.^21^, ^22^ Because of our interest in the possible functional consequences of HLA‐G DNA variants, we have performed extended sequencing to obtain information on combinations of the allele sequence and the 3′UTR region of HLA‐G. For this, we studied mother‐child pairs in a population that was mostly of European ancestry, since this approach enhances the reliability with which novel alleles and association between coding sequence and UTR are identified.

## 2 METHODS

### 2.1 Cohort

For an initial screen, 218 mother‐child pairs were selected as part of a Norwegian Pregnancy Biobank, after informed consent, as described in a previous publication.^23^ The study was approved by the Regional committee for Medical and Health Research Ethics in South‐Eastern Norway, and performed in accordance with the principles of the Helsinki Declaration. DNA was isolated from maternal and fetal EDTA plasma samples.^23^ Of the 218 mother‐child pairs included, 171 complete pairs were eventually available for which typing was obtained for the HLA‐G coding sequence.

### 2.2 Polymerase chain reaction (PCR)

PCR were carried out in a total volume of 15 μL consisting of 12.5 ng of genomic DNA, 0.3 pmol/μL forward primers, 0.3 pmol/μL reverse primers, 0.04 U of GoTaq (Promega), 0.2 mM dNTP, 4 mM MgCl_2_, 15 mM (NH_4_)_2_SO_4_, 50 mM Tris HCl, 0.05 mM EDTA, 0.01% gelatin, and 1 mM β‐mercaptoethanol. The PCR protocol consisted of an initial 2 minutes at 94°C, followed by 10 cycles consisting of 15 seconds at 94°C and 45 seconds at 68°C, and by 25 cycles consisting of 15 seconds at 94°C, 45 seconds at 64°C, and 30 seconds at 72°C. The program was finalized with 3 minutes at 72°C. For enzymatic cleanup, 15 μL of PCR product was subjected to 2 μL of ExoSAP‐IT (Applied Biosystems, Ottawa, Canada). Reactions were incubated for 30 minutes at 37°C and for 20 minutes at 80°C.

### 2.3 Sequencing

A sequencing kit (NGSgo‐AmpX HLA‐G; GenDx, Utrecht, The Netherlands) was used to identify HLA‐G alleles by determining polymorphisms in the coding sequence using next generation sequencing (NGS). Additionally, we have developed an in‐house sequence‐based typing (SBT) assay^24^ to sequence a 699/713‐bp fragment covering the HLA‐G 3′UTR of exon 8, starting just before the 14‐bp insertion/deletion and ending 591 bp downstream of it. Forward (GTG‐ATG‐GGC‐TGT‐TTA‐AAG‐TGT‐CAC‐C) and reverse (AGG‐GGA‐AGA‐GGT‐GTA‐GGG‐GTC‐TG) primers were from Sigma (St Louis MI). The reverse primer was extended with an M13 tail (GAC‐GTT‐GTA‐AAA‐CGA‐CGG‐CCA‐GT). Reaction tubes were sent to the Leiden Genome Technology Center and outsourced to Macrogen for Sanger sequencing. The 14‐bp insertion/deletion (rs371194629), +3003C/T (rs1707), +3010C/G (rs1710), +3027A/C (rs17179101), +3035C/T (rs17179108), +3142C/G (rs1063320), +3187A/G (rs9380142), +3196C/G (rs1610696), +3422C/T (rs17875408), +3496A/G (rs1233330), and +3509G/T (rs1611139) were identified to compose 3′UTR haplotypes.^18^ Information for UTR‐18 was acquired from another report.^25^ Interpretation was done with SBTengine (version 3.19) (GenDx) using a customized HLA‐G 3′UTR library.

**FIGURE 1:**
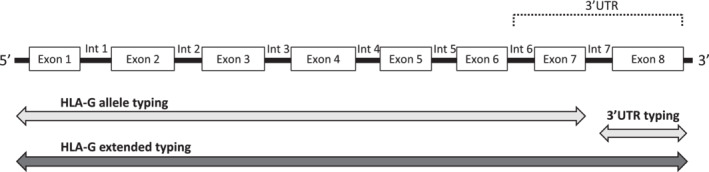
Full phasing over the whole HLA‐G gene sequence by extended next generation sequencing. HLA‐G allele typing was acquired by an NGSgo‐AmpX assay on an amplicon that starts at −57 and ends at +2727. A separate, in‐house developed sequencing assay targeted the 3′UTR region and generated an amplicon between +2940 and +3559. For a combined strategy of simultaneous assessment of the HLA‐G allele and 3′UTR haplotype, the forward primer (starting at −57) from the NGSgo‐AmpX assay was combined with the reverse primer (at 3559) from the in‐house developed sequencing assay

For a combined strategy of simultaneous assessment of HLA‐G alleles and 3′UTR haplotypes we performed extended typing, whereby the forward primer from the NGSgo‐AmpX assay was combined with the reverse primer from the in‐house developed SBT assay (see Figure 1). The technical protocol was similar to the NGS methodology that was followed to determine the HLA‐G alleles.

### 2.4 Linkage disequilibrium

To calculate the overall linkage disequilibrium (LD) between the HLA‐G coding sequence and the 3′UTR, PyPop 0.7.0 software (California)^26^ was used. The delta (*D*), relative delta (*D*′), and *P* value were used as measures of the strength of association. The *D*′ is a number between 0 and 1, with 1 meaning maximal association. A positive *D*, in combination with *D*′ approaching 1 and a significant *P* value, denotes high LD. For calculation of LD between different pairs of HLA‐G alleles and 3′UTR haplotypes, *D* was calculated along with the Pearson correlation coefficient (*r*), to determine the strength of the association.

## 3 RESULTS

From 171 mother‐child pairs the HLA‐G coding sequence, the HLA‐G 3′UTR, and the extended HLA‐G region were evaluated. Of the remaining 47 pairs, at least one of the assessments could not be adequately performed, mostly due to insufficient amount of DNA. Due to inheritance in the study cohort of family samples, the 171 complete mother‐child pairs gave opportunity to check reliability of the extended typing strategy and robustness of newly found alleles; provided that they were inherited by the child or not derived from the father.

For identification of haplotype frequencies we only report information from the mothers. The frequencies of the combined HLA‐G coding sequence and 3′UTR haplotype for the 342 available maternal chromosomes have been summarized in Table 1. We found nine new genotype variants of HLA‐G: six new HLA‐G alleles and three new 3′UTR haplotype variants (using IPD‐IMGT/HLA Database version 3.34). Of these, four HLA‐G alleles and one 3′UTR haplotype variant could be confirmed in the offspring, since they were inherited from mother to child. The sequences of the six new HLA‐G genotype variants have been registered in GenBank and the IPD‐IMGT/HLA Database, and their official sequence names, determined by the WHO Nomenclature Committee, have been incorporated in Table 1.

**TABLE 1:** Frequencies of extended HLA‐G haplotypes

Allele	3′UTR haplotype^a^	*N*	Frequency (%)
G*01:01:01:01	UTR‐1	112	32.7
G*01:01:02:01	UTR‐2	71	20.8
G*01:01:01:05	UTR‐4	49	14.3
G*01:04:01:01	UTR‐3	35	10.2
G*01:01:03:03	UTR‐7	19	5.6
G*01:06	UTR‐2	10	2.9
G*01:03:01:02	UTR‐5	11	3.2
G*01:01:22	UTR‐2	8	2.3
G*01:05N	UTR‐2	3	0.88
G*01:01:01:12 ^b^	UTR‐1	2	0.58
G*01:01:01:08	UTR‐1	2	0.58
G*01:04:04	UTR‐3	2	0.58
G*01:04:04	UTR‐3 new	2	0.58
G*01:01:01:13 ^c^	UTR‐6	2	0.58
G*01:01:01:04	UTR‐18	2	0.58
G*01:01:24 ^d^	UTR‐2	1	0.29
G*01:01:01:05	UTR‐1	1	0.29
G*01:01:02:04 ^e^	UTR‐2	1	0.29
G*01:06:01:02 ^f^	UTR‐2	1	0.29
G*01:23 ^g^	UTR‐3	1	0.29
G*01:01:01:01	UTR‐4	1	0.29
G*01:01:01:06	UTR‐4	1	0.29
G*01:01:01:06	UTR‐4 new	1	0.29
G*01:01:01:01	UTR‐6 new	1	0.29
G*01:01:01:04	UTR‐6	1	0.29
G*01:01:24 ^d^	UTR‐18	1	0.29
G*01:01:17	UTR‐2	1	0.29

*Note*: HLA‐G haplotypes from 342 maternal chromosomes have been depicted in order from highest to lowest frequency. The frequencies in the last column represent percentages of the total number of chromosomes analyzed.

a Haplotypes were composed based on singular polymorphisms in the 3′UTR region, according to the approach described previously.^18^ UTR‐18 is similar to UTR‐6, except for one position at +3227.^25^ UTR‐3 new is similar to UTR‐3 but with a G at position +3010. UTR‐4 new is similar to UTR‐4 but with a T at position +3092. UTR‐6 new is similar to UTR‐6 but with a T at position +3422, and could be verified in the offspring.

b This allele is a new variant of G*01:01:01:01, with a T instead of a C at +148 (intron 1). It was verified in the offspring. This novel allele has been included in the IPD‐IMGT/HLA Database (HLA26876).

c This allele is a new variant of G*01:01:01:04, with a T instead of a C at +1079 (exon 3). It was verified in the offspring. This novel allele has been included in the IPD‐IMGT/HLA Database (HLA26791).

d This allele is a new variant of G*01:01:22, with a T instead of a C at +456 (codon 85). It was verified in the offspring. This novel allele has been included in the IPD‐IMGT/HLA Database (HLA26786).

e This allele is a new variant of G*01:01:02:01, with a G instead of an A at +1179 (intron 3). It was verified in the offspring. This novel allele has been included in the IPD‐IMGT/HLA Database (HLA26990).

f This allele is a new variant of G*01:06, with a T instead of a C at +1921 (intron 4). Not inherited to the offspring. This novel allele has been included in the IPD‐IMGT/HLA Database (HLA27242).

g This allele is a new variant of G*01:04:01:01, with a T instead of a C at +37 (leader peptide, codon −12, Ser > Leu). Not inherited to the offspring. This novel allele has been included in the IPD‐IMGT/HLA Database (HLA26781).

For 684 chromosomes (342 maternal, 342 child) we could assess the consistency between the extended HLA‐G sequencing approach and the separate sequencing reactions that targeted either the coding sequence or the 3′UTR region. For 680 alleles (99.4%) consistent results were found. Four discrepancies were encountered. In two cases, one in a maternal DNA sample and one in a child DNA sample, extended sequencing gave a homozygous typing, whereas the separate allele typing and 3′UTR typing both gave a heterozygous result. In a third sample (mother), extended typing gave G*01:01:05, whereas allele typing gave G*01:04:01:01. In a fourth sample (child), extended typing of a non‐maternally inherited allele gave UTR‐4~G*01:01:05, whereas the separate typing methods gave UTR‐5/G*01:01:01:05. Extended typing was performed again on the four DNA samples showing discrepancy between the technical approaches, and this time the sequencing results matched between the extended sequencing approach and the two separate sequencing assays.

LD analysis was performed, and a strong overall association was found between the HLA‐G coding sequence and the 3′UTR (*D* = 0.066, *D*′ = 0.994, *P* < .0001). As shown in Table 2, particularly strong associations were found between HLA‐G*01:04 and UTR‐3, between HLA‐G*01:01:03 and UTR‐7, and between HLA‐G*01:03:01 and UTR‐5 (for each pair: positive *D* value and *r* = 1).

**TABLE 2:** Associations between pairs of HLA‐G alleles and 3′UTR haplotypes

Allele	UTR haplotype	*N* ^a^	Frequency (%)^b^	*D* ^c^	*r* ^c^
G*01:01:01	UTR‐1	117	100	0.159	0.613
G*01:01:02	UTR‐2	72	75.0	0.156	0.826
G*01:01:17/22/24 ^d^		10	10.4	0.019	0.246
G*01:05		3	3.1	0.006	0.139
G*01:06		11	11.5	0.024	0.273
G*01:04:01/04 ^e^	UTR‐3	40	100	0.107	1.000
G*01:01:01	UTR‐4	52	100	0.079	0.366
G*01:03:01	UTR‐5	11	100	0.032	1.000
G*01:01:01	UTR‐6	4	100	0.006	0.087
G*01:01:03	UTR‐7	19	100	0.054	1.000
G*01:01:01	UTR‐18	3	100	0.004	0.075

Allele	UTR haplotype	*N* ^a^	Frequency (%)^b^	*D* ^c^	*r* ^c^
G*01:01:01	UTR‐1	117	66.5	0.159	0.613
	UTR‐4	52	29.5	0.079	0.366
	UTR‐6	4	2.3	0.006	0.087
	UTR‐18	3	1.7	0.004	0.075
G*01:01:02	UTR‐2	72	100	0.156	0.826
G*01:01:03	UTR‐7	19	100	0.054	1.000
G*01:01:17/22/24 ^d^	UTR‐2	10	100	0.019	0.246
G*01:03:01	UTR‐5	11	100	0.032	1.000
G*01:04:01/04 ^e^	UTR‐3	40	100	0.107	1.000
G*01:05	UTR‐2	3	100	0.006	0.139
G*01:06	UTR‐2	11	100	0.024	0.273

a Total number of maternal chromosomes studied was 342.

b Frequency within the variant group that is denoted in the first column.

c *D* denotes delta, *r* denotes the correlation coefficient. *A* positive *D* value with *r* = 1 indicates strong linkage.

d G*01:01:24 is a novel allele that has been added to the IPD‐IMGT/HLA Database (HLA26786).

e This group includes G*01:23, which is a new variant of G*01:04:01:01. The novel allele has been added to the IPD‐IMGT/HLA Database (HLA26781).

## 4 DISCUSSION

We performed sequencing on mother‐child pairs to obtain the fully phased whole HLA‐G gene sequence, including information concerning both the allele and the 3′UTR region. In 99.4% of all alleles analyzed, the extended typing method gave consistent results as compared to the separate allele‐ and 3′UTR typing methods. Strong associations were found between HLA‐G*01:04 and UTR‐3, between HLA‐G*01:01:03 and UTR‐7, and between HLA‐G*01:03:01 and UTR‐5. We identified nine novel HLA‐G variants (either coding sequence or 3′UTR), five of which could be verified in the offspring.

A particularly strong association was found between the HLA‐G*01:04 allele and UTR‐3. Interestingly, this combination has been found to be related to low levels of soluble HLA‐G in the serum of lung transplant recipients and to adverse transplant outcome.^27^ In contrast to G*01:01 and G*01:03, G*01:04 exhibits an altered peptide anchor motif, and, when expressed on cells, it confers increased protection against cytotoxicity by natural killer cells.^12^ Recently, an NGS‐based approach was published to obtain information on the full HLA‐G gene, including the 5′UTR, the coding sequence, and the 3′UTR.^22^ In the current study a similar approach was followed, but here we had the advantage of investigating mother‐child combinations and the opportunity to internally validate several novel HLA‐G alleles and 3′UTR haplotype variants. Furthermore, we could validate consistency of the extended typing method in comparison to the separately typed coding and 3′UTR sequences. A limitation of our study is that the 5′UTR region was not included in the analysis.

The current findings in the Norwegian population for UTR‐1~HLA‐G*01:01 (34.2%) and UTR‐3~HLA‐G*01:04 (11.4%) match those described in a French population (33.6% and 15.5%, respectively).^19^ In other populations of European ancestry, frequency for both UTR‐3 and HLA‐G*01:04 was 8.5%.^10^ It should be noted, however, that the prevalence of combined allele/UTR haplotypes, as described in our study, may be different for other ethnic groups.

The four discrepancies that we encountered between the extended typing results and typing of the separate regions probably should be ascribed to an incidental imperfection in the extended typing technique. The first two cases may be due to a missed allele in the extended typing because of a flaw in PCR amplification. Case three and four may be due to contamination or carry over of the reaction. Repeating of the extended typing on the four DNA samples did give compatible results between extended typing and the two separate sequencing assays.

We have used the mother‐child pairs to identify novel alleles in the mother, and verify these in the child. Obviously, this could be done only in those cases were there was a considerable confidence that the particular allele was inherited from mother to child. In 18.7% of mother‐child pairs the child was either homozygous or the mother and child had similar genotypes, which complicated full segregation analysis. Furthermore, we did not have genetic information from the fathers.

The sequencing strategy provided for HLA‐G may be translated to classical HLA genes. The functional relevance of polymorphisms in the coding alleles of classical HLA genes is widely acknowledged, and availability of complementary UTR sequence information could provide an extra layer of information on the level of expression and on the functionality of the HLA molecules. Indeed, genetic variations in the UTR of HLA‐A are associated with expression.^28^ Variations in the 3′UTR of HLA‐C affect cell surface expression of the gene product and consequently viral control.^29^, ^30^ The expression level of HLA‐C also influences immunogenicity in incompatible transplantation conditions.^31^ Increasing knowledge of genetic variation in the UTR regions of both non‐classical and classical HLA genes would necessitate agreement on appropiate nomenclature and inclusion in the IPD‐IMGT/HLA Database.

In conclusion, we have performed extended sequencing of the HLA‐G gene and have described both allele coding sequence/UTR combinations and novel genotype variants of the HLA‐G gene.

## CONFLICT OF INTEREST

The authors have declared no conflicting interests.

## Data Availability

The research data in this article may be shared.
